# Comparative Analysis of Next- and Third-Generation Sequencing Platforms for Chikungunya Virus Whole-Genome Sequencing Using a Lineage-Inclusive Primer Set During the 2025 Foshan Outbreak

**DOI:** 10.3390/tropicalmed11020044

**Published:** 2026-02-05

**Authors:** Penghui Jia, Xiao Cong, Chang Zhang, Zhe Liu, Xiaofang Peng, Juan Su, Qiqi Tan, Shen Huang, Changyun Sun, Xin Zhang, Baisheng Li

**Affiliations:** 1 Institute of Pathogenic Microbiology, Guangdong Provincial Center for Disease Control and Prevention, Guangzhou 511400, China; jiaph2025@163.com (P.J.); congx3@mail2.sysu.edu.cn (X.C.);; 2 Guangdong Provincial Key Laboratory of Pathogen Detection for Emerging Infectious Disease Response, Guangdong Workstation for Emerging Infectious Disease Control and Prevention, Chinese Academy of Medical Sciences, Guangzhou 511430, China; 3 Organ Transplant Center, The First Affiliated Hospital, Sun Yat-sen University, Guangzhou 510062, China; 4 Guangdong Provincial Institute of Public Health, Guangzhou 511430, China

**Keywords:** chikungunya virus, whole-genome sequencing, genomic surveillance, outbreak investigation, lineage-inclusive primer set, phylogenetics

## Abstract

Chikungunya virus (CHIKV) poses an increasing global public health threat, as evidenced by the significant 2025 Foshan outbreak in China. Rapid, whole-genome sequencing (WGS) is critical for outbreak response but is challenged by primer mismatches across diverse lineages and a lack of direct sequencing platform comparisons. To address this, we developed a novel lineage-inclusive primer set and performed parallel WGS on 24 clinical samples from the outbreak using both Illumina (NGS) and Oxford Nanopore Technologies (TGS) platforms. Our lineage-inclusive primer set successfully amplified full-length CHIKV genomes across all samples. Comparisons revealed that Illumina NGS provided higher raw read accuracy, while Nanopore TGS achieved more complete coverage of terminal UTRs with a faster turnaround time. Crucially, after polishing, variant calls between the two platforms were 100% concordant. Phylogenetic analysis was consistent with a single introduction event, with all outbreak isolates forming a monophyletic clade within the ECSA lineage most closely related to contemporaneous strains from Réunion Island. This study validates a lineage-inclusive amplicon-based sequencing strategy and demonstrates that NGS and TGS offer complementary advantages. When integrated, they provide a robust framework for real-time genomic surveillance, enhancing preparedness and guiding public health interventions against CHIKV.

## 1. Introduction

Chikungunya virus (CHIKV) is a mosquito-borne, positive-sense single-stranded RNA virus belonging to the family Togaviridae and genus *Alphavirus* [1]. Since its first isolation in Tanzania in 1952, CHIKV has been responsible for recurrent and increasingly frequent outbreaks across Africa, Asia, Europe, and the Americas, facilitated by globalization, climate change, and expanding distributions of the competent vectors *Aedes aegypti* and *Aedes albopictus* [2,3]. Clinical manifestations typically include acute fever and severe polyarthralgia; however, up to 40–60% of patients may develop chronic, debilitating joint symptoms that persist for months to years, imposing a substantial long-term socioeconomic burden [4,5,6].

Driven by globalization, urbanization, and climate change, CHIKV infection exhibits a rapid capacity for geographical expansion, posing a significant threat to newly affected regions. The recent 2025 Foshan outbreak in China exemplifies this persistent threat. In July 2025, an unexpected and rapidly expanding chikungunya fever outbreak occurred in Foshan City, Guangdong Province, China—one of the most significant CHIKV epidemics in the region in recent years. Within a few weeks, cases increased sharply and spread across multiple districts [1,5,7]. Prior to this outbreak, China had documented only imported CHIKV cases in 2008, followed by several localized outbreaks in Guangdong [8], Zhejiang [9], and Yunnan Provinces [10] between 2010 and 2019, all linked to imported infections. Therefore, real-time genomic characterization is essential for identifying the viral origin, tracking transmission routes, detecting adaptive mutations, and providing timely evidence to guide public health interventions.

The ~11.8 kb CHIKV genome encodes four nonstructural proteins (nsP1-nsP4) essential for viral replication and five structural proteins (C, E3, E2, 6K, and E1), whose genetic variability contributes to viral adaptation, transmissibility, and epidemic potential [4]. Of particular epidemiological importance is the E1-A226V mutation, which enhances viral fitness in *Ae. albopictus* and has been implicated in several major global outbreaks [11,12,13]. Despite the recognized value of genomic surveillance, obtaining complete and accurate CHIKV whole-genome sequences remains challenging. Key obstacles include low viral loads in clinical samples, genetic variation among circulating strains, and uneven genome coverage. This is particularly problematic in the 5′ and 3′ untranslated regions (UTRs), which are essential for viral replication and regulation.

Currently, next-generation sequencing (NGS) and third-generation sequencing (TGS) represent the two primary platforms used in viral genomics [14]. NGS platforms, such as Illumina, offer high-throughput sequencing with exceptional base accuracy, making them the gold standard for reliable single-nucleotide variant (SNV) detection [15]. However, their short read length often results in incomplete assembly of repetitive or structurally complex regions, leading to gaps in UTRs and other regulatory elements. By contrast, TGS platforms such as Oxford Nanopore Technologies (ONT) generate long reads capable of spanning entire amplicons or even complete genomes, providing superior assembly continuity and resolving structural features and haplotypes [16]. Yet, TGS typically exhibits higher raw error rates, requiring robust downstream polishing algorithms to ensure variant-calling accuracy. Thus, choosing between NGS and TGS for outbreak response involves trade-offs among accuracy, completeness, speed, and cost.

A frequently overlooked determinant of sequencing success is the quality of genome amplification. Clinical samples often contain insufficient viral RNA for direct sequencing, necessitating targeted amplification using lineage-inclusive primer sets. Existing primer schemes may fail to amplify diverse CHIKV strains uniformly, leading to coverage bias or incomplete genomes that compromise downstream evolutionary analyses. Moreover, although both NGS and TGS have been individually applied to CHIKV sequencing, a systematic head-to-head comparison using a unified, optimized primer set has not been conducted. Consequently, data are lacking on the relative performance of these platforms in real-world outbreak samples—particularly regarding coverage uniformity, variant concordance, and phylogenetic resolution.

To address these gaps, we designed a novel lineage-inclusive CHIKV primer set targeting highly conserved genomic regions and evaluated its amplification efficiency in clinical samples collected during the 2025 Foshan outbreak. Using this unified primer scheme, we performed parallel WGS on 24 confirmed CHIKV-positive serum samples using both Illumina NGS and Oxford Nanopore TGS. We systematically compared platform performance in terms of genome coverage, sequencing depth, accuracy, variant concordance, and phylogenetic reconstruction. Additionally, we analyzed the molecular characteristics and evolutionary relationships of the outbreak strain to infer transmission dynamics and potential origin. This study therefore provides a validated, practical framework for CHIKV genomic surveillance and offers critical insights into the complementary roles of NGS and TGS in outbreak response.

## 2. Materials and Methods

### 2.1. Sample Collection and Clinical Information

A total of 24 laboratory-confirmed CHIKV-positive serum samples were collected during the chikungunya fever outbreak in July 2025 in Foshan City, Guangdong Province, China. Samples were obtained from three hospitals in Beijiao Town, Shunde District: Chencun Hospital (*n* = 12), Beijiao Hospital (*n* = 7), and Heyou Hospital (*n* = 5). All patients presented with typical symptoms, including acute fever and arthralgia. For each case, demographic and clinical data—such as age, sex, date of symptom onset, and sampling date—were recorded. The collected serum samples were residual specimens from routine clinical tests such as complete blood counts, with associated clinical information provided by the hospitals. We extracted RNA from the serum and performed RT-PCR to confirm CHIKV infection, recording the corresponding cycle threshold (Ct) values as indicators of viral load. Samples spanning a wide range of Ct values were intentionally included to evaluate sequencing performance across different viral titers.

### 2.2. Design of a Lineage-Inclusive CHIKV Primer Set

Complete CHIKV genomes representing the three major phylogenetic lineages—East/Central/South African (ECSA), West African, and Asian (Accessions: PV685524.1, PV685664.1, PV685706.1)—were retrieved from the NCBI GenBank database. Genomes containing >1% ambiguous bases or incomplete 5′/3′ untranslated regions (UTRs) were excluded. Conserved regions across lineages were identified and used as the template for primer design. A set of 10 overlapping primer pairs was designed using PrimalScheme v3.0.2 (https://primalscheme.com/ (accessed on 16 July 2025)), generating amplicons of approximately 1200 bp with ~200 bp overlap between adjacent fragments to ensure robust whole-genome coverage (~11.8 kb). Primer sequences and genomic positions are listed in Table A1.

### 2.3. RT-PCR Amplification and Amplicon Generation

Viral RNA was extracted from serum samples. cDNA was synthesized from the extracted RNA using the LunaScript RT SuperMix Kit (NEB, Ipswich, MA, USA) under the following conditions: 25 °C for 2 min, 50 °C for 10 min, and 95 °C for 1 min for enzyme inactivation. The resulting cDNA was used for multiplex long-range PCR to amplify the complete CHIKV genome. A tiling-amplicon strategy employing two non-overlapping primer pools was used to generate ~1.2 kb overlapping fragments. PCR amplification was performed with Q5 High-Fidelity 2× Master Mix using the following cycling program: 98 °C for 30 s; 35 cycles of 95 °C for 15 s and 65 °C for 5 min. PCR products were purified using 0.5× AMPure XP beads (Beckman Coulter, Brea, CA, USA) and eluted in nuclease-free water prior to quantification.

### 2.4. Illumina Library Preparation and Sequencing

Illumina libraries were constructed from purified amplicons using the Illumina DNA Prep Kit (Illumina, San Diego, CA, USA). The workflow included enzymatic fragmentation and size selection, end-repair and A-tailing, and ligation of unique dual indexes (UD Indexes Set A) during a limited-cycle PCR. The indexed libraries were then purified, quantified, normalized, and pooled at equimolar concentrations. Pooled libraries were denatured and diluted to the recommended loading concentration and sequenced on the Illumina MiniSeq platform (Illumina, San Diego, CA, USA) using a single-end configuration (1 × 150 bp). Base calling was performed through the iterative incorporation and imaging of fluorescently labeled nucleotides during bridge amplification on the flow cell.

### 2.5. Oxford Nanopore Library Preparation and Sequencing

For Oxford Nanopore sequencing, ~1.2 kb amplicons were prepared using the Native Barcoding Kit 24 (SQK-NBD114.24, Oxford Nanopore Technologies plc, Oxford, UK). The workflow consisted of three enzymatic steps: (1) end-repair and dA-tailing, (2) ligation of unique native barcodes using Blunt/TA Ligase Master Mix (NEB, Ipswich, MA, USA), and (3) pooling and adapter ligation with Quick T4 DNA Ligase (NEB, Ipswich, MA, USA). The final barcoded library was purified with AMPure XP beads (Beckman Coulter, Brea, CA, USA) and loaded onto a primed R10.4.1 flow cell for sequencing on the GridION or MinION platform.

### 2.6. Bioinformatic Analysis of Illumina NGS and Nanopore TGS Data

Sequencing data from both Illumina NGS and Oxford Nanopore TGS platforms were processed using standardized reference-guided workflows. Initial taxonomic confirmation of CHIKV reads was performed using Kraken2 (Illumina: v2.1.2; Nanopore: v2.1.6). Raw sequencing data of Illumina NGS was processed using fastp (v0.23.2) with the following key filtering steps: (i) automatic adapter trimming; (ii) removal of reads containing more than 10% unidentified bases (N); (iii) truncation of low-quality bases from the 5′ and 3′ ends (quality threshold < 20); and (iv) discarding of reads shorter than 50 bp after trimming. Only reads passing these filters were used for subsequent alignment and variant calling. Illumina reads were aligned to the CHIKV reference genome (strain S27b03, GenBank: PV685524.1) using BWA v0.7.17, whereas Nanopore long reads were aligned using minimap2 v2.17. Variant calling was conducted using FreeBayes v1.3.5 for Illumina data and Medaka v1.7.3 for Nanopore data, followed by low-quality variant filtering with bcftools (Illumina: v1.12; Nanopore: v1.19). Nanopore consensus sequences underwent an additional polishing step using Racon to correct residual base-calling errors. For both platforms, final consensus genomes were reconstructed through reference-guided assembly and subsequently genotyped using the Chikungunya Typing Tool (https://www.genomedetective.com (accessed on 23 December 2025)). Multiple sequence alignment was performed with MAFFT v7.453, and Neighbor-Joining phylogenetic trees were inferred using MEGA11 software, with bootstrap support assessed from 1000 replicates. Phylogenies were visualized using the Interactive Tree of Life (iTOL, https://itol.embl.de (accessed on 23 December 2025)).

### 2.7. Comparative Evaluation of Sequencing Platform Performance

To directly compare the performance of Illumina NGS and Oxford Nanopore TGS, sequencing data from the same 24 samples were processed in parallel using unified reference genomes and standardized evaluation criteria. Genome coverage, per-base depth, and coverage uniformity were calculated from aligned BAM files. Read quality and length distributions were assessed using Illumina Q20/Q30 scores and Nanopore median Q-scores and N50 values. A site-by-site comparison revealed that the set of high-confidence SNV positions and the genotypes at those positions were identical between platforms for all samples. Phylogenetic consistency was evaluated by comparing Neighbor-Joining trees generated independently from NGS and TGS consensus genomes. Together, these metrics provided an integrated evaluation of the strengths and limitations of both sequencing technologies for CHIKV whole-genome analysis.

## 3. Results

### 3.1. Clinical Characteristics of CHIKV-Positive Samples

A total of 24 CHIKV-positive serum samples were collected during the July 2025 Foshan outbreak. All patients presented with acute febrile illness accompanied by arthralgia. Ct values ranged from 20 to 29, representing a wide viral-load distribution suitable for evaluating sequencing performance across titers. Table 1 summarizes demographic and clinical information for all cases.

### 3.2. Validation of Lineage-Inclusive Amplicon Amplification

**Figure 1:**
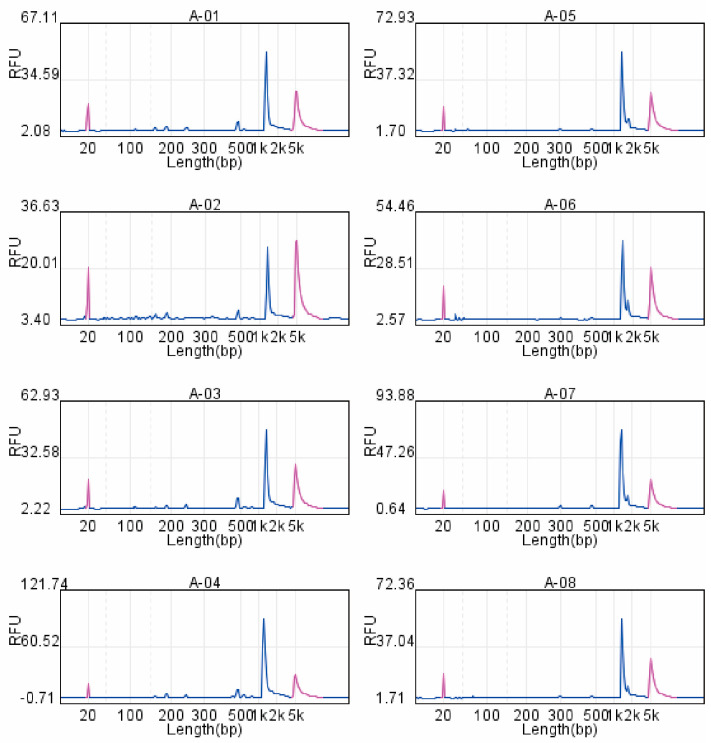
Capillary electrophoresis analysis of lineage-inclusive CHIKV primers using clinical samples. Panels A-01 to A-04 correspond to amplifications with Primer Pool 1 (P1, containing 10 upstream primers), while Panels A-05 to A-08 correspond to amplifications with Primer Pool 2 (P2, containing 10 downstream primers). The *x*-axis represents the fragment length in base pairs (bp), and the y-axis shows the fluorescence intensity in Relative Fluorescence Units (RFU).

To validate the performance of the newly designed lineage-inclusive CHIKV primer set, amplicons generated from four representative clinical samples (Samples 1–4) using two primer pools were first quantified by Qubit fluorometry, yielding DNA concentrations of 30–70 ng/μL. Subsequently, capillary electrophoresis was performed. The 20 individual primers were divided into two multiplex pools. Primer Pool 1 (P1) contained the 10 forward primers, and Primer Pool 2 (P2) contained the 10 reverse primers corresponding to each of the 10 tiling amplicons. All amplification reactions produced a single, dominant peak at the expected size of approximately 1.2 kb (indicated by the dashed vertical line). The absence of secondary peaks confirms the specific and efficient amplification of the target tiling fragments, with no detectable primer-dimer artifacts or non-specific products (Figure 1).

### 3.3. Illumina NGS Achieves High-Depth and Uniform Coverage of the CHIKV Genome

**Figure 2:**
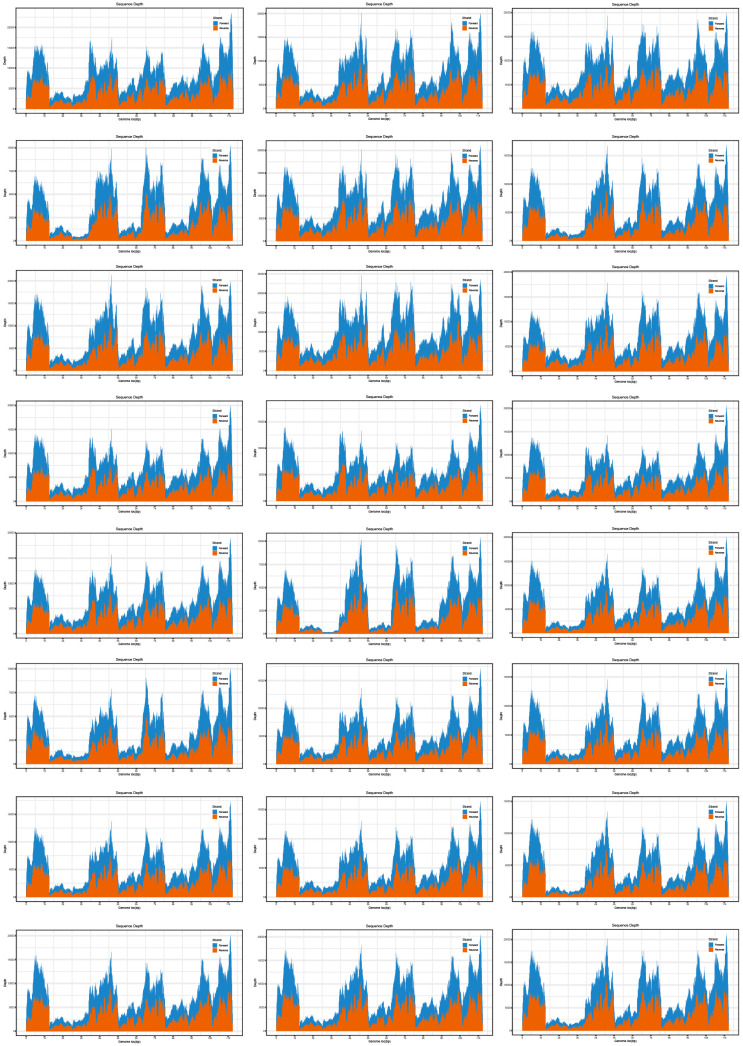
Sequencing depth profiles of NGS. Sequencing depth maps were generated from next-generation sequencing (NGS) data. Each panel represents a distinct sequence. The *x*-axis denotes the genomic position of the assembled viral, while the y-axis represents the sequencing depth (coverage). The blue and orange shaded areas likely indicate the depth of forward and reverse reads, respectively. The varying heights reflect heterogeneity in coverage depth across the genome.

Illumina sequencing generated a median of 0.90 million high-quality reads per sample (mean: 0.91 million; interquartile range: 0.31 million), with high base quality (mean Q20: 95.77%; Q30: 93.40%). Alignment to the reference genome (S27b03, including both coding and non-coding UTRs) yielded a mean depth of >6000× across all 24 samples, with every sample achieving complete 100% coverage and no gaps in any genomic region. The unequivocally confirms the robustness of the sequencing and the primer set’s comprehensive representation of the viral genome. The sequencing depth profile, illustrated in Figure 2, demonstrates exceptionally uniform read enrichment across the entire coding sequence (CDS), underscoring the platform’s reliability for achieving comprehensive genomic coverage. Detailed per-sample sequencing metrics are provided in Table A2.

### 3.4. Sequencing Performance of Oxford Nanopore TGS

**Figure 3:**
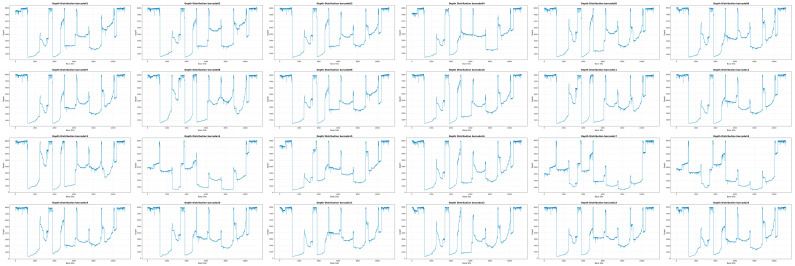
Electropherogram profiles of third-generation sequencing data of TGS. The figure presents a panel of raw sequencing traces (electropherograms) generated by a third-generation sequencing platform (Oxford Nanopore Technologies). Each horizontal trace represents a continuous long read from the sequencing run. The *x*-axis of each electropherogram represents the time or base position in the read, while the *y*-axis corresponds to the signal intensity measured during the nucleotide incorporation event.

Nanopore GridION sequencing (20 h run time) generated 200–500 Mb per sample, with a median read length of ~1000 bp and median Q-score ~Q16. Coverage heatmaps (Figure 3) demonstrated complete coverage (100%) with depths ranging from 500× to 8000× across samples. Barcode-specific depth plots confirmed uniform long-read tiling coverage without terminal drop-outs.

### 3.5. NGS and TGS Demonstrate Complementary Performance Characteristics in Coverage and Accuracy

A comparative evaluation of Illumina NGS and Oxford Nanopore TGS demonstrated complementary performance characteristics across the 24 CHIKV genomes. Illumina sequencing yielded more uniform per-base depth, reflected by lower coverage variation, whereas Nanopore achieved more complete coverage of the terminal UTRs and other repetitive regions. Read-quality metrics showed a mean Illumina Q30 exceeding 93%, while Nanopore achieved a median Q-score of approximately Q16 with a read-length N50 of ~1.1 kb. High-confidence SNVs (depth > 100×; allele frequency > 5%) were consistently detected in all samples, and notably, the variant sets derived from both platforms were fully concordant after polishing of Nanopore reads with Medaka and Racon, confirming that consensus-level accuracy was equivalent across platforms. Error-profile analysis indicated fewer indels in Illumina data, whereas Nanopore raw reads exhibited higher insertion and deletion frequencies; however, these errors were largely corrected during polishing, resulting in comparable substitution patterns between platforms. Workflow comparisons showed that Nanopore offered a shorter overall turnaround time (~12–15 h from amplicon to consensus genome), whereas Illumina required ~25–30 h but provided higher intrinsic base accuracy. Together, these metrics highlight the complementary strengths of both technologies and underscore their combined value for rapid and accurate CHIKV whole-genome sequencing.

### 3.6. Concordance in Variant Calling and Identification of Key Mutations

**Figure 4:**
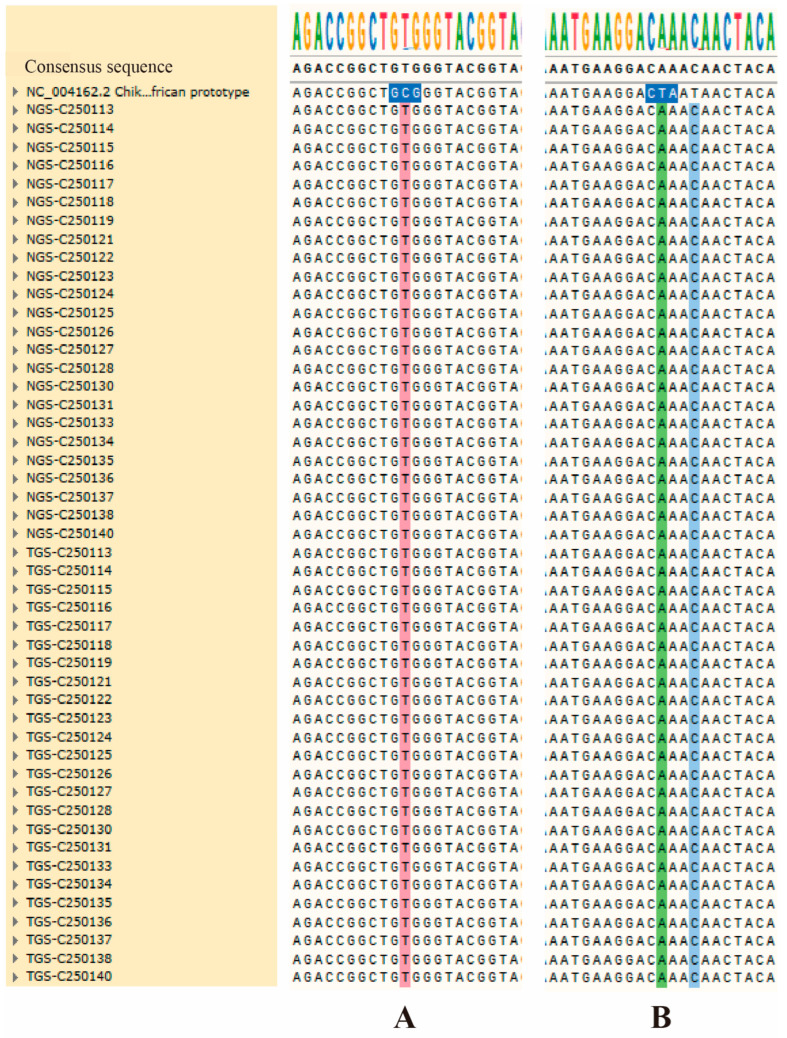
Universal presence of the E1-A226V and E2-L210Q mutation in outbreak CHIKV sequences. (**A**) Nucleotide sequence alignment of 24 outbreak-derived CHIKV isolates with the reference strain NC_004162.2, targeting the E1-226 codon; all isolates uniformly display a nucleotide substitution at position 10,669 of the E1 gene, which encodes the A226V amino acid substitution. (**B**) Nucleotide sequence alignment of the same 24 outbreak-derived CHIKV isolates with the reference strain NC_004162.2, targeting the E2-210 codon; all isolates uniformly exhibit a nucleotide substitution at position 9169 of the E2 gene, which results in the L210Q amino acid substitution.

High-confidence single-nucleotide variants (SNVs) (depth > 100×, AF > 5%) were jointly analyzed across the 24 CHIKV genomes sequenced using Illumina NGS and Oxford Nanopore TGS. Variant calls were fully concordant between the two platforms after polishing of Nanopore reads, and no platform-specific discrepancies were observed for true biological variants. Two lineage-associated adaptive mutations—E1-A226V and E2-L210Q—were detected in 100% (*n* = 24) of the samples. Multiple sequence alignments confirmed that all consensus genomes shared the characteristic alanine-to-valine substitution at residue 226 of the E1 protein (Figure 4A) as well as the leucine-to-glutamine substitution at E2 position 210 (Figure 4B), both of which have been associated with enhanced transmission by *Ae. albopictus* and increased epidemic potential.

Beyond these genotype-defining mutations, several additional SNVs were identified across the dataset, including 3879 C → T (24/24 samples), 9874 C → T (8/24), and 2959 G → A (2/24). These lower-frequency variants were not located in known adaptation-associated regions but were consistently detected in both NGS and polished TGS data. Conversely, the 9099 A → T substitution was observed in three Nanopore raw assemblies but was determined to be an artifact upon depth inspection: coverage at this position exceeded 4900× and 7200× in representative samples C250136 and C250137, respectively, with negligible support for the alternate allele. Therefore, 9099 A → T was excluded as a true biological variant. The concordance statement pertained exclusively to SNVs; no high-confidence indels were identified in any sample, consistent with the close evolutionary relationship to the reference strain S27b03 and the acute nature of the outbreak. A complete summary of all detected SNVs is provided in Table 2. Table 2 provides a summary of mutation loci identified by both NGS and TGS. In this table, the columns represent the mutation positions within the reference genome. The notation “C/T”, for example, indicates a substitution where the reference base C is altered to T in the sample. The rows, conversely, correspond to the sample ID.

This combined analysis highlights the strong cross-platform robustness in variant detection and confirms that the Foshan 2025 outbreak strain carries the canonical E1-A226V and E2-L210Q mutations characteristic of the epidemic ECSA lineage.

### 3.7. Phylogenetic Analysis and Outbreak Origin

**Figure 5:**
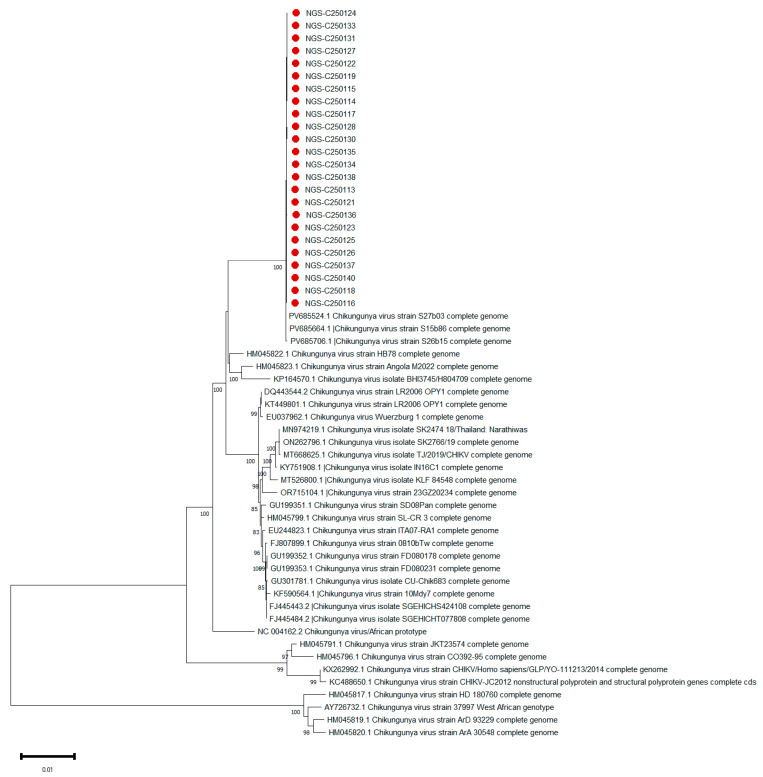
Phylogenetic reconstruction of 24 Foshan CHIKV genomes and reference global strains. Neighbor-joining tree based on full-genome sequences, showing the evolutionary placement of the 24 Foshan CHIKV genomes (red circles) among global reference strains (black labels). Bootstrap values ≥ 80% (from 1000 replicates) are indicated at the nodes. The scale bar represents genetic distance. The analysis confirms that the Foshan outbreak viruses form a monophyletic cluster within the ECSA lineage.

To determine the genetic relationship and potential origin of the Foshan outbreak strains, a phylogenetic analysis was conducted using neighbor-joining methods based on the complete genome sequences of the 24 isolates obtained in this study, alongside a curated set of globally representative CHIKV reference sequences encompassing all major genotypes (ECSA, Asian, and West African) and contemporary strains from the 2025 Réunion Island outbreak (Accessions: PV685524.1, PV685664.1, PV685706.1). The neighbor-joining trees reconstructed from both NGS- and TGS-derived consensus sequences yielded identical topologies with strong nodal support (bootstrap values > 90%), demonstrating the robustness and platform-independence of our phylogenetic inferences. The analysis revealed that all 24 Foshan isolates formed a distinct, well-supported monophyletic clade (bootstrap value = 100%) with no discernible genetic diversity, suggesting a scenario consistent with a single introduction event followed by clonal expansion within the local population (Figure 5). Crucially, this Foshan clade robustly nested within the East/Central/South African (ECSA) genotype, and demonstrated the closest evolutionary relationship with the contemporaneous CHIKV sequences from Réunion Island, forming a shared subclade with strong statistical support (bootstrap value = 100%). This high-degree genetic relatedness, evidenced by the minimal number of nucleotide substitutions between the Foshan and Réunion sequences, provides molecular evidence compatible with a recent transmission chain. The phylogenetic data are consistent with the hypothesis that the outbreak may have originated from a single introduction event linked to the ongoing epidemic in Réunion Island.

## 4. Discussion

Our study successfully addresses the need for a unified sequencing approach to characterize CHIKV outbreaks by developing a lineage-inclusive primer set and conducting a systematic comparison of NGS and TGS platforms during the 2025 Foshan outbreak. By integrating Illumina NGS and Oxford Nanopore TGS workflows under a unified amplicon-based strategy, we directly compared the performance of these platforms for whole-genome sequencing of CHIKV. Through comprehensive evaluation of genome coverage, sequencing depth, mutation profiling, and phylogenetic resolution, we characterized the strengths and limitations of each method, providing meaningful insights for CHIKV genomic surveillance and outbreak response. Our study directly addresses these gaps by not only delivering a validated lineage-inclusive primer set but also providing a definitive, head-to-head comparison of NGS and TGS platforms using a unified amplification strategy.

A significant contribution of this study is the development of a 10-pair primer scheme that uniformly amplified all genomic regions, including conserved and variable segments across ECSA, Asian, and West African lineages. Capillary electrophoresis verification confirmed clean, monodisperse ~1.2 kb peaks without detectable non-specific amplification, while Qubit quantification demonstrated consistently high DNA yields suitable for downstream library preparation. This finding is significant because primer bias and lineage-restricted amplification remain key obstacles in CHIKV genomic research, particularly during outbreaks in newly affected regions where circulating strains may differ from those used in earlier primer designs [17,18,19,20]. These results highlight the primer set’s strong lineage inclusivity and amplification robustness even at higher Ct values, addressing a common technical barrier in CHIKV sequencing where primer mismatches or low viral loads often lead to incomplete genomes. The development of this primer set therefore provides a valuable tool for both routine molecular surveillance and rapid outbreak investigation.

Parallel sequencing using Illumina and Nanopore platforms clearly demonstrated complementary strengths. Illumina produced highly accurate reads with exceptional base quality (mean Q30 >93%), uniform per-base depth, and minimal indel noise, reaffirming its status as the gold standard for precise SNV detection [21]. In contrast, Nanopore sequencing provided near-complete coverage of the genome—including 5′ and 3′ UTRs that typically exhibit drop-offs in NGS—while maintaining rapid turnaround times (~12–15 h from amplicon to consensus). The ability of Nanopore long reads to bridge structurally complex regions enabled gap-free genome assemblies that are particularly advantageous in outbreak settings requiring rapid molecular characterization [22,23]. Notably, the variant concordance analysis revealed 100% agreement between polished TGS consensus genomes and NGS variant calls, underscoring the effectiveness of Nanopore polishing pipelines in mitigating raw error rates and supporting its reliability when appropriate analytical workflows are applied.

Mutation analysis further highlighted the genomic features associated with the 2025 Foshan outbreak strain. All 24 samples uniformly carried the epidemiologically significant E1-A226V and E2-L210Q mutations, which are known to enhance viral fitness and transmissibility in *Ae. albopictus* [7,24]. This result aligns with CHIKV evolutionary patterns observed in previous large-scale outbreaks across the Indian Ocean region and Southeast Asia [25,26]. Together, these observations demonstrate both the precision of the analytical pipeline and the value of integrating multiple sequencing platforms for robust mutation detection.

Phylogenetic analyses based on both Illumina and Nanopore consensus sequences yielded identical topologies, reflecting strong platform consistency. All Foshan isolates clustered tightly into a monophyletic clade nested within the ECSA lineage, showing the closest evolutionary affinity to contemporaneous strains from the Réunion Island epidemic [11,27,28]. The minimal genetic diversity observed among Foshan genomes is highly consistent with a single introduction event followed by rapid local transmission, mirroring the epidemiological characteristics of the outbreak. These findings reinforce the critical role of genomic surveillance in tracing viral introductions and reconstructing transmission chains, especially in regions like southern China where *Ae. albopictus* is abundant and repeated arboviral introductions have been documented over the last decade [8].

This study has several strengths, including a comprehensive cross-platform evaluation, robust primer design, and high-quality genomic data. Nevertheless, some limitations should be acknowledged. First, while the tiling-amplicon approach employed here ensures high genome completeness, it inherently limits the detection of structural variants or recombination events that fall outside the primer binding sites. For a comprehensive view of such genomic alterations, metagenomic or hybrid-capture sequencing would be required in future studies. Second, while Nanopore sequencing offers rapid results, its accuracy remains dependent on coverage depth and subsequent polishing; improvements in base-calling algorithms and pore chemistry may further enhance reliability in future investigations. Finally, the study focused on a single outbreak; integrating vector-derived sequences and broader temporal sampling could provide deeper insights into the ecological and evolutionary dynamics of CHIKV transmission in Guangdong. Additionally, while the primer set demonstrated robust ‘lineage-inclusive’ amplification of the major CHIKV lineages in this study, its comprehensive applicability can be further enhanced. Future work will involve designing and validating primers against a broader geographical and temporal diversity of genomes to progress towards a truly ‘pan-lineage’ assay.

Overall, this study underscores the value of integrating an optimized primer system with both second- and third-generation sequencing platforms to achieve rapid, high-quality genomic surveillance during an active outbreak. The methodological framework developed here integrates robust amplification, cross-platform sequence evaluation, and comprehensive phylogenetic analysis. This approach can be readily implemented in public health laboratories to facilitate timely molecular characterization of emerging arboviral pathogens. As CHIKV continues to expand into new geographic regions and adapt to diverse ecological environments, the availability of reliable genomic tools such as those described in this study will be essential for early detection, risk assessment, and the effective management of future outbreaks.

## 5. Conclusions

In conclusion, our study successfully addresses critical challenges in the genomic surveillance of CHIKV outbreaks. We have developed and validated a novel lineage-inclusive, primer set that enables robust and unbiased whole-genome amplification of diverse CHIKV strains, thereby overcoming a significant technical barrier in frontline molecular epidemiology. Through a systematic head-to-head comparison, we demonstrated that Illumina NGS and Oxford Nanopore TGS, when used within this unified amplicon-based framework, offer complementary strengths. NGS remains the gold standard for base-level accuracy, while TGS provides superior speed and completeness, particularly in genomic regions problematic for short reads. Importantly, after rigorous bioinformatic polishing, consensus sequences from both platforms achieved 100% variant concordance, establishing the reliability of TGS for rapid outbreak investigation. Applied to the 2025 Foshan outbreak, this integrated approach precisely characterized the outbreak strain as a monophyletic cluster of the ECSA lineage bearing key adaptive mutations, and pinpointed its origin to the contemporaneous Réunion Island epidemic. The methodological framework and comparative insights provided here offer a practical, scalable, and effective solution for public health laboratories worldwide to conduct real-time genomic surveillance of CHIKV, thereby enhancing preparedness and response capabilities against this re-emerging arboviral threat.

## Figures and Tables

**Table 1:** Characteristics of the 24 CHIKV-positive patients.

ID	Gender	Age	Ct	Date of Symptom Onset	Date of Sample Collection
C250113	Male	72	20	12 July 2025	12 July 2025
C250114	Female	37	24	11 July 2025	13 July 2025
C250115	Male	66	22	12 July 2025	13 July 2025
C250116	Male	19	27	12 July 2025	13 July 2025
C250117	Male	32	24	11 July 2025	13 July 2025
C250118	Female	66	27	7 July 2025	13 July 2025
C250119	Female	9	26	12 July 2025	13 July 2025
C250121	Male	79	23	12 July 2025	13 July 2025
C250122	Male	62	22	13 July 2025	13 July 2025
C250123	Male	68	22	12 July 2025	13 July 2025
C250124	Female	84	23	12 July 2025	13 July 2025
C250125	Female	55	24	12 July 2025	13 July 2025
C250126	Male	42	24	12 July 2025	13 July 2025
C250127	Male	48	29	11 July 2025	13 July 2025
C250128	Female	21	28	12 July 2025	13 July 2025
C250130	Female	48	27	12 July 2025	13 July 2025
C250131	Female	77	25	11 July 2025	13 July 2025
C250133	Female	59	28	12 July 2025	13 July 2025
C250134	Male	10	27	13 July 2025	13 July 2025
C250135	Female	8	26	13 July 2025	13 July 2025
C250136	Male	43	28	12 July 2025	13 July 2025
C250137	Male	8	27	12 July 2025	13 July 2025
C250138	Female	20	27	12 July 2025	13 July 2025
C250140	Male	13	28	12 July 2025	13 July 2025

Demographics, real-time PCR Ct values, and dates of symptom onset and sample collection are shown for each patient. The cohort (13 males, 11 females; age range: 8–84 years) had Ct values between 20 and 29. Most samples (*n* = 20) were collected on 13 July 2025, shortly after symptom onset.

**Table 2:** Concordance of variant calls between NGS and TGS across 24 CHIKV genomes.

ID/Sites	420	688	1557	2860	2904	2959	3481	3879	3897	5312	7446	9099	9449	9874	10,488
C250113	-	-	-	-	-	-	-	C/T	-	-	-	-	-	-	-
	-	-	-	-	-	-	-	C/T	-	-	-	-	-	-	-
C250114	-	-	-	-	-	-	-	C/T	-	-	-	-	-	C/T	-
	-	-	-	-	-	-	-	C/T	-	-	-	-	-	C/T	-
C250115	-	-	-	-	-	-	-	C/T	-	-	-	-	-	C/T	-
	-	-	-	-	-	-	-	C/T	-	-	-	-	-	C/T	-
C250116	-	-	-	-	-	-	-	C/T	C/T	-	-	-	-	-	-
	-	-	-	-	-	-	-	C/T	C/T	-	-	-	-	-	-
C250117	-	-	-	-	-	-	T/C	C/T	-	T/C	-	-	-	-	-
	-	-	-	-	-	-	T/C	C/T	-	C/T	-	-	-	-	-
C250118	-	-	-	-	-	-	-	C/T	-	-	-	-	-	-	-
	-	-	-	-	-	-	-	C/T	-	-	-	-	-	-	-
C250119	-	-	-	-	-	-	-	C/T	-	-	-	-	-	C/T	-
	-	-	-	-	-	-	-	C/T	-	-	-	-	-	C/T	-
C250121	-	-	-	-	-	-	-	C/T	-	-	-	-	-	-	-
	-	-	-	-	-	-	-	C/T	-	-	-	-	-	-	-
C250122	-	-	-	-	-	-	-	C/T	-	-	-	-	-	C/T	-
	-	-	-	-	-	-	-	C/T	-	-	-	-	-	C/T	-
C250123	-	-	-	-	-	-	-	C/T	-	-	-	-	-	-	-
	-	-	-	-	-	-	-	C/T	-	-	-	-	-	-	-
C250124	A/C	-	-	G/A	-	-	-	C/T	-	-	-	-	-	C/T	-
	-	-	-	G/A	-	-	-	C/T	-	-	-	-	-	C/T	-
C250125	-	-	-	-	-	-	-	C/T	-	-	-	-	-	-	-
	-	-	-	-	-	-	-	C/T	-	-	-	-	-	-	-
C250126	-	-	-	-	-	-	-	C/T	-	-	-	-	-	-	-
	-	-	-	-	-	-	-	C/T	-	-	-	-	-	-	-
C250127	-	-	-	-	-	-	-	C/T	-	-	-	-	-	C/T	-
	-	-	-	-	-	-	-	C/T	-	-	-	-	-	C/T	-
C250128	-	-	-	-	-	G/A	-	C/T	-	-	-	-	-	-	-
	-	-	-	-	-	G/A	-	C/T	-	-	-	-	-	-	-
C250130	-	-	-	-	-	G/A	-	C/T	-	-	-	-	-	-	-
	-	-	-	-	-	G/A	-	C/T	-	-	-	-	-	-	-
C250131	-	-	-	-	-	-	-	C/T	-	-	-	-	-	C/T	-
	-	-	-	-	-	-	-	C/T	-	-	-	-	-	C/T	-
C250133	-	-	-	-	-	-	-	C/T	-	-	-	-	-	C/T	-
	-	-	-	-	-	-	-	C/T	-	-	-	-	-	-	-
C250134	-	-	-	-	G/A	-	-	C/T	-	-	-	-	-	-	-
	-	-	-	-	G/A	-	-	C/T	-	-	-	A/T	-	-	-
C250135	-	-	T/C	-	-	-	-	C/T	-	-	-	-	-	-	-
	-	-	T/C	-	-	-	-	C/T	-	-	-	-	-	-	-
C250136	-	G/A	-	-	-	-	-	C/T	-	-	-	-	C/T	-	C/T
	-	G/A	-	-	-	-	-	C/T	-	-	-	A/T	C/T	-	C/T
C250137	-	-	-	-	-	-	-	C/T	-	-	-	-	-	-	-
	-	-	-	-	-	-	-	C/T	-	-	-	A/T	-	-	-
C250138	-	-	-	-	-	-	-	C/T	-	-	T/C	-	-	-	-
	-	-	-	-	-	-	-	C/T	-	-	T/C	-	-	-	-
C250140	-	-	-	-	-	-	-	C/T	-	-	-	-	-	-	-
	-	-	-	-	-	-	-	C/T	-	-	-	-	-	-	-

The table displays nucleotide variants detected at 15 genomic sites for each of the 24 outbreak sequences. Data from NGS (top of each cell) and TGS (bottom of each cell) are shown for direct comparison. The absence of a variant is indicated by a dash (-).

## Data Availability

The complete genome sequences generated in this study are in the process of being deposited in the GenBank database.

## Abbreviations

The following abbreviations are used in this manuscript:

CHIKV	Chikungunya virus
NGS	Next-generation sequencing
TGS	Third-generation sequencing
ONT	Oxford Nanopore Technologies
WGS	Whole-genome sequencing
ECSA	East/Central/South African
SNV	Single-nucleotide variant
UTR	Untranslated region
RT-PCR	Reverse transcription polymerase chain reaction
Ct	Cycle threshold
CDS	Coding sequence

## Appendix A

**Table A1:** Primers used for amplification of the full-length CHIKV genome.

Name	Sites	Sequence
LEFT_1	8–30	GTGAGACACACGTAGCCTACCA
RIGHT_1	1328–1353	TGCTTTTTGAATGCCCATAGACAGC
LEFT_2	1284–1307	AGATGAAAAACTCCTGGGGGTCA
RIGHT_2	2604–2626	CAGTGTACACCGCCTGGAGATA
LEFT_3	2565–2590	TTACAATCACAACATCTGCACCCAA
RIGHT_3	3885–3907	TCCCAATACGCAGATGACTCGT
LEFT_4	3797–3818	AAATGCTCGGGGGTGACTCAT
RIGHT_4	5116–5136	AGTATCTCGCCGTCAACGCT
LEFT_5	5069–5091	AGGAAGCGAGTACGGCTATGTC
RIGHT_5	6389–6412	TGTTATCCTGATAGGGCTGGCAG
LEFT_6	6318–6341	GGACTCAGCAGTATTCAACGTGG
RIGHT_6	7637–7659	TCTGGGCCTGATGACCTGGATA
LEFT_7	7590–7614	TTTTACAACAGGAGGTACCAGCCT
RIGHT_7	8910–8931	GTGAAATGGGTGCGTGCATGA
LEFT_8	8877–8899	GTGGGATTCACTGACGGTAGGA
RIGHT_8	10,197–10,224	GCTGTAGTCAGGTAGATTTTTGTCCTT
LEFT_9	10,165–10,185	CGTACGTGAAATGCTGCGGT
RIGHT_9	11,485–11,513	CCTACATACAATGTGTCTCTTAGGGGAC
LEFT_10	10,475–10,498	CATTGTGGGGCCAATGTCTTCAG
RIGHT_10	11,790–11,823	ATATTAAAAACAAAATAACATCTCCTACGTCCC

The table lists the names, genomic binding sites (relative to reference strain NC_004162.2), and nucleotide sequences of the ten overlapping primer pairs (LEFT_1/RIGHT_1 to LEFT_10/RIGHT_10) used to generate amplicons covering the complete coding region of the Chikungunya virus genome.

**Table A2:** Summary of Illumina MiniSeq sequencing metrics and genome coverage for 24 clinical CHIKV samples.

ID	Reads	% Reads Identified (PF)	Total Data Volume (Mb)	Q20 (%)	Q30 (%)	Average Sequencing Depth
C250113	1,087,729	4.61	101.82	96	93	8168.0
C250114	1,132,029	4.81	103.1	97	95	7814.4
C250115	1,197,112	5.08	109.43	96	94	8533.1
C250116	526,196	2.24	48.6	95	92	3607.2
C250117	1,235,424	5.24	112.93	96	94	8825.8
C250118	839,145	3.57	78.07	96	93	5983.1
C250119	1,143,369	4.86	105.02	96	94	8165.5
C250121	1,490,474	6.33	135.25	96	94	10678.9
C250122	953,481	4.03	89.67	96	94	7097.4
C250123	919,858	3.90	86.44	96	94	6758.4
C250124	829,318	3.51	77.86	96	94	6130.0
C250125	866,515	3.68	81.41	95	93	6345.3
C250126	901,988	3.82	84.61	96	94	6700.1
C250127	522,734	2.24	48.63	95	93	3301.1
C250128	903,634	3.84	84.89	96	93	6512.1
C250130	484,677	2.06	44.1	96	93	3346.0
C250131	782,767	3.32	73.59	96	93	5674.2
C250133	773,226	3.29	72.73	96	94	5630.7
C250134	796,468	3.39	74.59	95	93	5745.3
C250135	736,375	3.12	69.35	95	93	5418.2
C250136	705,394	3.15	66.0	95	93	4994.1
C250137	947,998	4.03	89.27	96	93	7041.6
C250138	1,015,585	4.31	95.36	96	94	7607.9
C250140	1,092,526	4.66	100.53	96	94	7627.7

Summary of Illumina MiniSeq sequencing performance for the 24 clinical samples from the 2025 Foshan CHIKV outbreak. **Reads:** Number of high-quality reads per sample after demultiplexing and stringent quality filtering (see Methods). **Total Data Volume**: Total yield of sequencing data per sample in megabases (Mb). **Q20/Q30 (%):** Percentage of bases with a Phred quality score greater than 20 or 30, respectively. **Average Sequencing Depth (×):** Mean coverage depth across the complete CHIKV reference genome (S27, NC_004162.2) after mapping. All samples achieved 100% genome coverage with the lineage-inclusive primer set. For the dataset (*n* = 24), the median number of reads per sample was 0.84 million (IQR: 0.73–1.04 million).
